# Long Non-Coding RNA SNHG3 Promotes the Progression of Cholangiocarcinoma by Regulating the miR-151a-3p/STAT5a Axis

**DOI:** 10.5152/tjg.2024.24140

**Published:** 2024-12-01

**Authors:** Xiaoping Wei, Dongyun Cun, Danping Yang, Qianyao Yi, Daguang Tian

**Affiliations:** Department of Hepatobiliary Surgery, The Second Affiliated Hospital of Kunming Medical University, Kunming, Yunnan, China

**Keywords:** SNHG3, cholangiocarcinoma, miR-151a-3p, STAT5a

## Abstract

**Background/Aims:**

Studies have shown the significance of long non-coding RNAs (lncRNAs) in the development of malignant tumors, including cholangiocarcinoma (CCA). However, the molecular mechanisms through which the lncRNA SNHG3 contributes to CCA development remain unknown. Therefore, the purpose of this work was to investigate SNHG3’s role and possible processes in CCA.

**Materials and Methods:**

CCK-8, TUNEL, wound healing, and transwell assays were performed to evaluate the viability, apoptosis, migration, and invasion of CCA cells, respectively. Dual-luciferase reporter and RNA pull-down assays were conducted to verify the relationship between SNHG3 and miR-151a-3p and that between STAT5a and miR-151a-3p.

**Results:**

SNHG3 and STAT5a were considerably upregulated and miR-151a-3p was downregulated in CCA tissues and cells. SNHG3 knockdown suppressed the proliferation, apoptosis, migration, and invasive ability of HUCC-T1 cells. Mechanistically, SNHG3 directly targeted miR-151a-3p to promote the development of CCA. Treatment with a miR-151a-3p inhibitor reversed the effects of SNHG3 knockdown on the aggressive behavior of HUCC-T1 cells. Furthermore, STAT5a knockdown counteracted the effects of inhibition of SNHG3 and miR-151a-3p on the aggressive behavior of CAA.

**Conclusion:**

SNHG3 promotes CCA progression via the miR-151a-3p/STAT5a axis, providing novel insights into the clinical treatment of CCA.

**Main Points**

- SNHG3 specifically targets miR-151a-3p in CCA.
- miR-151a-3p directly targets STAT5a in CCA.
- SNHG3 promotes CCA growth and metastasis through miR-151a-3p/STAT5a.

## Introduction

Cholangiocarcinoma (CCA) is an extremely aggressive and life-threatening malignant adenoma originating from the epithelial lining of bile ducts.^1^ Based on the location of the tumor, CCA is classified as extrahepatic cholangiocarcinoma (EHCC) and intrahepatic cholangiocarcinoma (ICC).^2^ Although advances in tumor treatment have improved the outcomes of individuals with CCA to a certain extent, the long-term survival rate remains low owing to late diagnosis, tumor invasion and metastasis, and drug resistance.^3^ Targeted gene therapy, which involves the introduction of exogenous genetic material into specific cells to regulate gene expression, has emerged as a promising strategy for inhibiting CCA progression.^4^ The pathological mechanisms underlying the development of CCA as well as the risk factors and genes associated with CCA remain elusive. Therefore, identifying novel biomarkers and target genes is necessary for efficient diagnosis, prevention, and management of CCA.

SNHG3 has been shown to participate in the progression of various tumors.^5-8^ Zhang et al ^6^ reported that SNHG3 was upregulated in hepatocellular carcinoma and this upregulation was associated with tumor size. In osteosarcoma, SNHG3 expression is positively associated with tumor growth, whereas knockdown of SNHG3 remarkably reduces the viability and clone-forming ability of tumor cells. Tian et al ^9^ reported the upregulation of SNHG3 in clinical CCA tissues, providing the first direct evidence of the relationship between SNHG3 and CCA. Altogether, these studies indicate that SNHG3 is a promising prognostic biomarker for CCA.

SNHG3 plays a crucial role in various carcinomas; however, its function in CCA still unknown. Therefore, the purpose of this study was to look into the function and mechanism of SNHG3 in CCA. Our findings revealed a novel axis comprising SNHG3, miR-151a-3p, and STAT5a that promoted the growth and metastasis of CCA. This study not only improves the understanding of complex gene regulation in CCA but also introduces novel avenues for the diagnosis and treatment of CCA.

## Materials and Methods

### Clinical Samples

Thirty paired tumor tissues and tumor-adjacent normal tissues were collected from patients with CCA who underwent surgical resection in Kunming Medical University between September 2022 and September 2024. The tumor-adjacent normal tissues refer to tissues located 2 cm away from the lesion and are commonly used as a control in cancer studies. All patients signed an informed consent form before participating in the study. This study was approved by the Ethics Committee of Kunming Medical University (approval number: SHEN-PJ-KE-2022-21; date: January 25, 2022).

### Immunohistochemical Staining

Immunohistochemical (IHC) analysis was performed to assess STAT5a expression in clinical tissue samples. The slices were exposed to an anti-STAT5a antibody (1:500; ab32043, Abcam, USA) incubation at 4°C overnight. The following day, the slices were treated with a secondary antibody at 25°C for 1 hour.

### Cell Culture

H69, HuH-28, TFK-1, HUCC-T1, and QBC-939 were acquired from the Cell Bank of Chinese Academy of Sciences (Shanghai, China). All cells were cultured in RPMI-1640 (Hyclone, USA) with 10% FBS (Hyclone, USA) at 37°C with 5% CO_2_.

### Cell Transfection

For inhibition of SNHG3 and STAT5a, small interfering RNAs (siRNAs) targeting SNHG3 (si-SNHG3) and STAT5a (si-STAT5a) and the corresponding negative control (si-NC) were synthesized by GenePharma. The sequences for cell transfection were listed in Supplementary Table 1. HUCC-T1 cells were transfected with 50 nM of sequences using Lipofectamine 2000 (Invitrogen, USA) and incubated for 24 hours.

### CCK-8 Assay

HUCC-T1 cells were planted in a 96-well plate with 1 × 10^5^ cells/mL. At 0, 24, 48, and 72 hours, the cells were treated with 100 μL of the CCK-8 (Beyotime, China) at 37°C for 2 hours. Subsequently, a microplate reader was utilized to measure the optical density at 450 nm.

### TUNEL Staining

HUCC-T1 cells were fixed with 4% polyformaldehyde (Beyotime, China) and added with 50 μL of the TUNEL (Beyotime, China) at 37°C for 60 minutes. Apoptotic cells with green fluorescence were detected using a fluorescence microscope (Olympus, Japan).

### Wound Healing Assay

After 48 hours of culture, a 1-mL sterile tube was used to create a scratch on the cell monolayer. At 0 and 48 hours, the width of the scratch was measured using a light microscope (Olympus, Japan).

### Transwell Assay

HUCC-T1 cells were resuspended in RPMI-1640 medium (Hyclone, USA) and either pre-treated with Matrigel (BD Biosciences, USA) or not injected into the top transwell chamber for cell migration and invasion. Additionally, the lower chamber was filled with 20% FBS. After 24 hours, the migratory or invasive cells were observed under a light microscope (Olympus, Japan).

### Subcellular Fractionation Assay

Subcellular fractionation was performed using the PARIS™ Kit (Invitrogen, USA) according to the specification. Quantitative real-time polymerase chain reaction (qRT-PCR) was employed to assess SNHG3 expression.

### Dual-Luciferase Reporter Assay

The binding site of SNHG3 was mutated from 5’-GAAUAUGCAUGUUACCAGUCUAG-3’ to 5’-GAAUUCGCUUGAAACGUCAGAUG-3’, and that of STAT5a was mutated from 5’-UUGGGCUUCAUUCAAGUCUAU-3’ to 5’-UUGGGCUUCAUUCAUCAGAUU-3’. The wild-type or mutant sequences of SNHG3 or STAT5a were cloned into pGL3-Firefly-Renilla plasmids to establish luciferase reporter plasmids encoding wild-type SNHG3 (SNHG3-WT), mutant SNHG3 (SNHG3-MUT), wild-type STAT5a (STAT5a-WT), and mutant STAT5a (STAT5a-MUT). HUCC-T1 cells were co-transfected with 100 pmol of a miR-151a-3p mimic or inhibitor and 0.5 g of a luciferase reporter plasmid. Subsequently, the activity was determined using a dual-luciferase reporter assay system (Promega, Madison, WI, USA).

### RNA Pull-down Assay

The Pierce Magnetic RNA-Protein Pull-Down Kit (Thermo, USA) was used to carry out the RNA pull-down experiment. Magnetic beads were added to samples to obtain RNA–protein complexes, and RNA enrichment was evaluated via qRT-PCR.

### Quantitative Real-Time Polymerase Chain Reaction

qRT-PCR was performed using SYBR Premix Ex TaqII (Takara, Otsu, Shiga, Japan) according to the manufacturer’s instructions. The primer sequences are listed in Supplementary Table 2. The 2^–ΔΔCt^ method was employed to determine the mRNA expression in comparison to that of β-actin or U6.

### Western Blotting

Total protein was isolated using RIPA (Beyond, China). Equal amounts of proteins (50 μg) from each group were subjected to SDS-PAGE. The membrane was incubated with primary antibodies at 4°C for 12 hours: anti-PCNA (1:1000; ab152112, Abcam, USA), anti-Ki-67 (1:1000; ab92742, Abcam, USA), anti-STAT5a (1:1000; ab32043, Abcam, USA), anti-β-actin (ab8226; 1:5000, Abcam, USA) antibodies, and incubated with the secondary antibody for 1 hour.The signals were detected using a Pierce® ECL western blotting substrate (Pierce, Thermo Fisher Scientific, Waltham, MA, USA).

### Statistical Analysis

All data were expressed as the mean ± SEM from at least 3 independent experiments. The unpaired Student’s *t-*test was used to compare data between 2 groups, whereas 1-way ANOVA followed by Bonferroni correction was used to compare data among 3 or more groups. All statistical tests were performed using the GraphPad 8.0.2 software (GraphPad Software, La Jolla, USA), with *P*-values of <.05 indicating statistically significant differences.

## Results

### SNHG3 and STAT5a were Upregulated and miR-151a-3p was Downregulated in Cholangiocarcinoma Tissues

**Figure 1.**
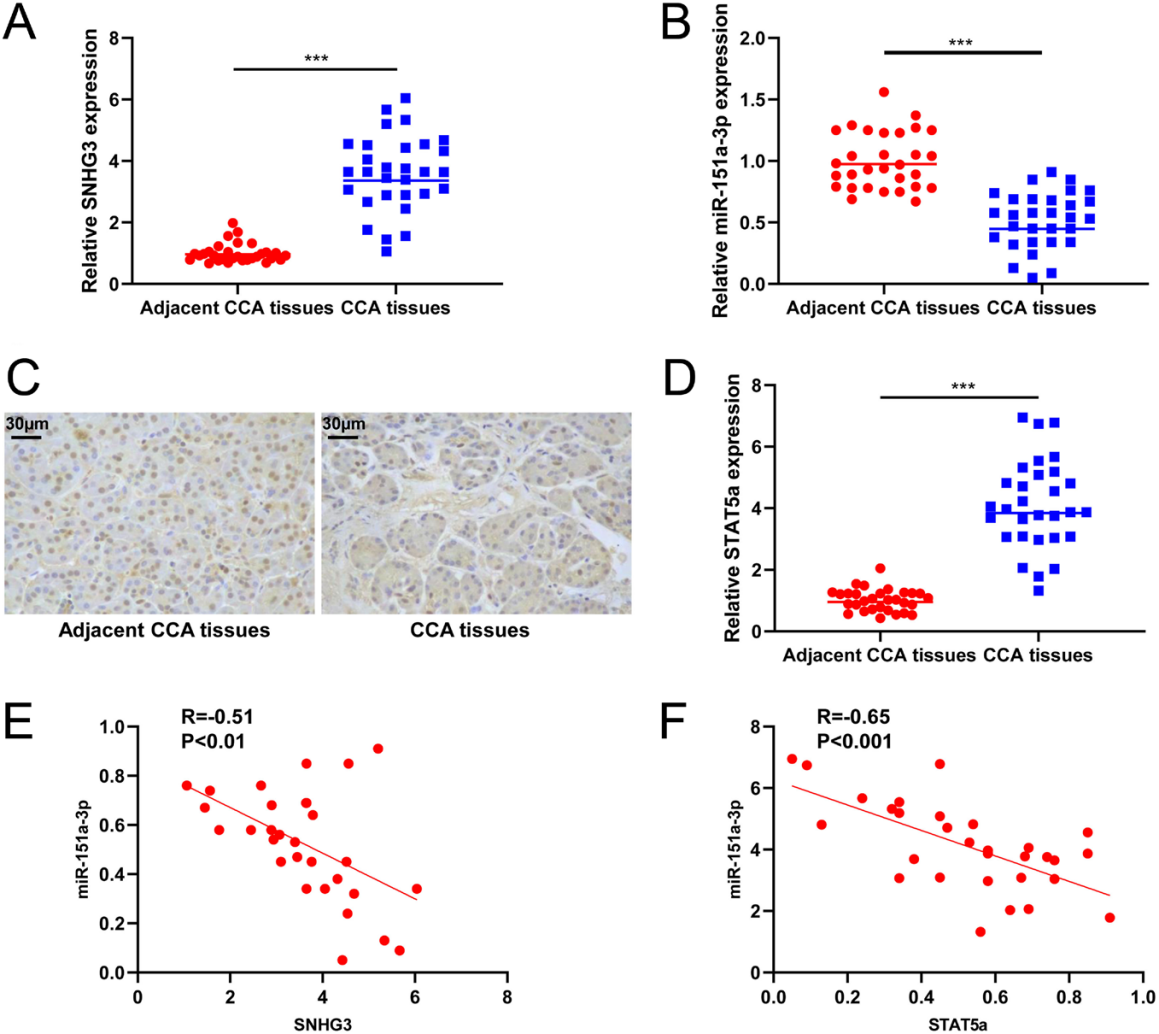
SNHG3 and STAT5a were upregulated and miR-151a-3p was downregulated in CCA tissues. (A) The level of SNHG3 in CCA tissues and adjacent CCA tissues was detected by qRT-PCR assay (n = 30). (B) The level of miR-151a-3p in CCA tissues and adjacent CCA tissues was detected by qRT-PCR assay (n = 30). (C) IHC was used to assess the level of STAT5a in CCA tissues and adjacent CCA tissues (magnification: ×100). (D) The level of STAT5a in CCA tissues and adjacent CCA tissues was detected by qRT-PCR assay (n = 30). (E) Pearson correlation analysis between SNHG3 and miR-151a-3p in CCA tissues. (F) Pearson correlation analysis between STAT5a and miR-151a-3p in CCA tissues. ^***^P < .001 vs. adjacent CCA tissues. CCA, cholangiocarcinoma.

Quantitative real-time polymerase chain reaction showed that SNHG3 was increased (*P* < .001, Figure 1A) and miR-151a-3p was downregulated in CCA tissues (*P* < .001, Figure 1B). In addition, IHC analysis and qRT-PCR showed that STAT5a was substantially elevated in CCA tissues (*P* < .001, Figure 1C-D). miR-151a-3p expression was negatively correlated with SNHG3 (*P* < .01, Figure 1E) and STAT5a (*P* < .001, Figure 1F). Furthermore, the correlation between SNHG3 level and clinicopathological features was analyzed. The results demonstrated that SNHG3 expression was significantly correlated with tumor differentiation, TNM stage, and lymph node invasion and metastasis (Table 1).

### Inhibition of SNHG3 Suppressed the Aggressive Behavior of Cholangiocarcinoma Cells

**Figure 2.**
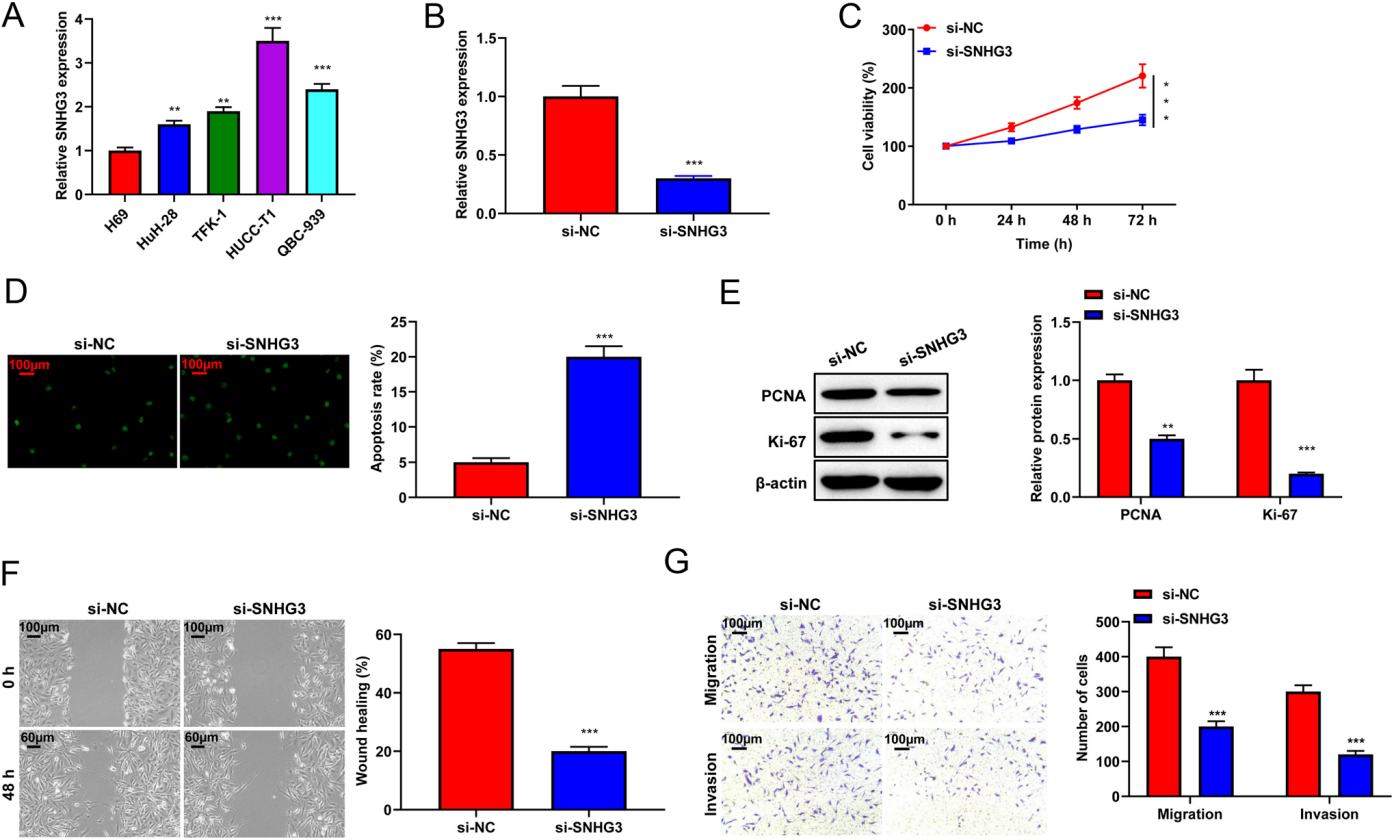
Inhibition of SNHG3 suppressed the aggressive behavior of CCA cells. (A) The level of SNHG3 in CCA cell lines (Huh-28, TFK-1, HUCC-T1, and QBC-939) and normal human cholangiocyte cell lines (H69) was measured by qRT-PCR assay. (B) HUCC-T1 cells were transfected with si-NC or si-SNHG3 for 24 hours. Quantitative real-time polymerase chain reaction assay confirmed the transfection efficiency of si-SNHG3 in HUCC-T1 cells. (C) CCK-8 assay was used to assess HUCC-T1 cells viability. (D) TUNEL was performed to detect HUCC-T1 cells apoptosis. (E) The protein expression levels of PCNA and Ki-67 were measured by Western blot assay. (F) Wound-healing assay was carried out to investigate HUCC-T1 cell migration (magnification: ×100). (G) Transwell assay was used to elucidate HUCC-T1 cell migration and invasion (magnification: × 100). *P < .05, **P < .01,***P < .001 vs. H69 or si-NC. CCA, cholangiocarcinoma; NC, negative control.

SNHG3 level was higher in Huh-28, TFK-1, HUCC-T1, and QBC-939 cells than in H69 cells (*P* < .001, Figure 2A). As HUCC-T1 cells exhibited the highest expression of SNHG3, they were employed in the following studies. To investigate SNHG3’s cellular role in CCA, si-SNHG3 was transfected into HUCC-T1 cells. Quantitative real-time polymerase chain reaction validated the successful inhibition of SNHG3 in HUCC-T1 cells (*P* < .001, Figure 2B). Inhibition of SNHG3 markedly suppressed the viability of HUCC-T1 cells (*P* < .05, Figure 2C) and increased their apoptotic rate (*P* < .001, Figure 2D). Western blotting showed that inhibition of SNHG3 significantly reduced the protein expression of PCNA and Ki-67 in HUCC-T1 cells (*P* < .001, Figure 2E). In addition, inhibition of SNHG3 effectively suppressed HUCC-T1 cells migration and invasion (*P* < .001, Figure 2F-G).

### SNHG3 Directly Targeted miR-151a-3p in Cholangiocarcinoma

**Figure 3.**
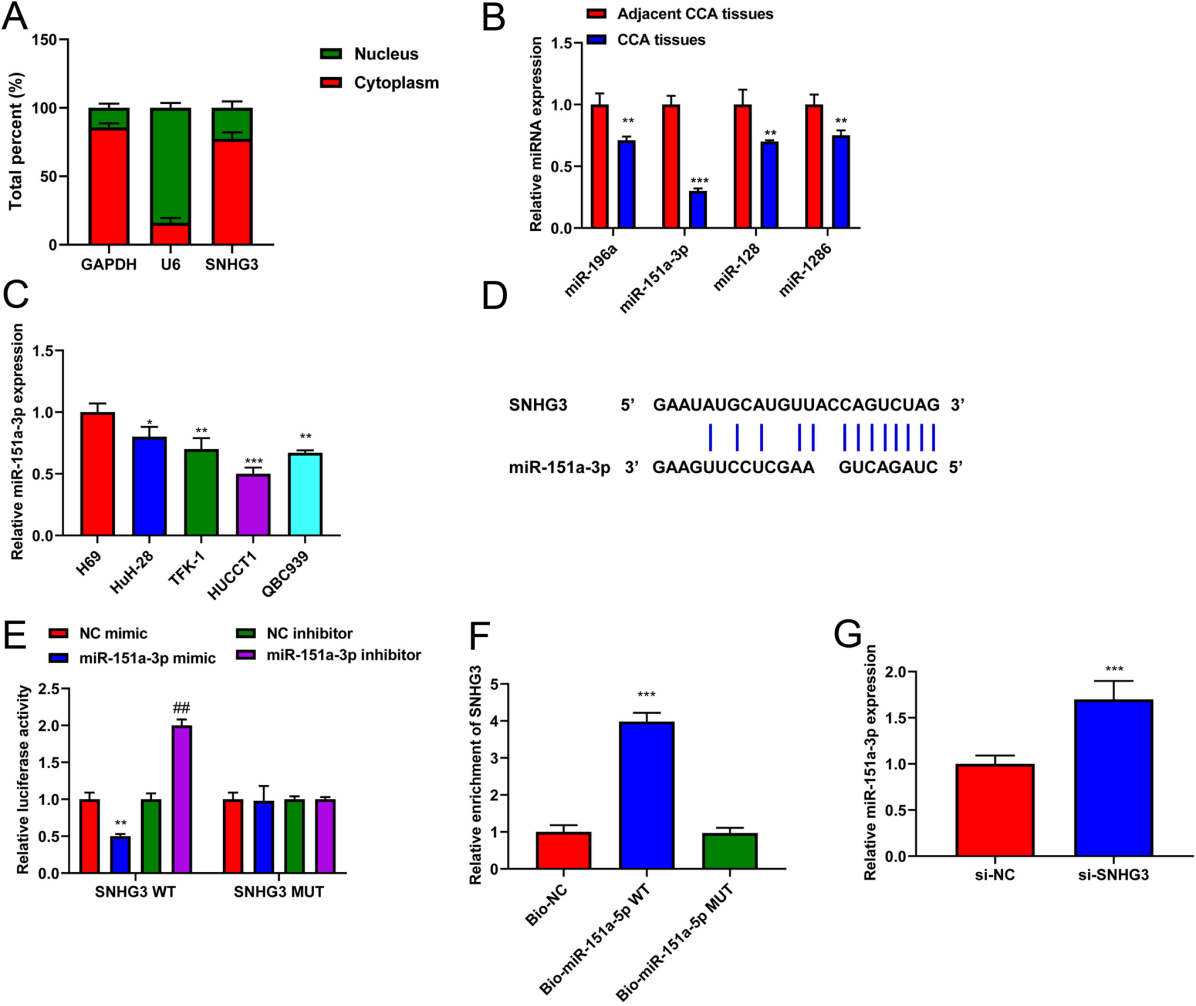
SNHG3 directly targeted miR-151a-3p in CCA. (A) Subcellular fractionation was performed to analyze the distribution of SNHG3 in CCA cells. (B) The levels of miR-196a, miR-151a-3p, miR-128, and miR-1286 in CCA tissues and adjacent CCA tissues were detected by qRT-PCR assay. (C) The level of miR-151a-3p in CCA cell lines (Huh-28, TFK-1, HUCC-T1, and QBC-939) and normal human cholangiocyte cell lines (H69) was measured by qRT-PCR assay. (D) The promising binding sites between SNHG3 and miR-151a-3p were predicted by Starbase. (E) Dual-luciferase reporter assay was carried out to prove the relationship between SNHG3 and miR-151a-3p. (F) RNA pull down assay was used to detect the binding situation between SNHG3 and miR-151a-3p. (G) The level of miR-151a-3p in HUCC-T1 cells with si-NC and si-SNHG3 transfection was detected by qRT-PCR assay. *P < .05, **P < .01, ***P < .001 vs. adjacent CCA tissues or H69 or NC mimic; ##*P* < .01 vs. NC inhibitor. CCA, cholangiocarcinoma; NC, negative control.

Subcellular fractionation was performed to analyze the distribution of SNHG3 in CCA cells. SNHG3 was present in the cytoplasm, where it may have a ceRNA-like role (Figure 3A). Then, the PubMed database was used to identify the target miRNAs of SNHG3 and the Starbase tool was utilized to anticipate the binding sites of SNHG3 in target miRNAs. Among the identified target miRNAs, miR-196a, miR-151a-3p, miR-128, and miR-1286 were selected owing to their important roles in tumor development.^10-13^ Quantitative real-time polymerase chain reaction showed that miR-196a, miR-151a-3p, miR-128, and miR-1286 were decreased (*P* < .01) and miR-151a-3p had the maximum downregulation (*P* < .001) in CCA tissues (Figure 3B). Consistently, miR-151a-3p was remarkably low in CCA cell lines (*P* < .001, Figure 3C). The binding sites of between SNHG3 and miR-151a-3p are shown in Figure 3D. As anticipated, treatment with a miR-151a-3p mimic decreased the luciferase activity of cells transfected with SNHG3-WT (*P* < .01, Figure 3E). Consistently, the biotinylated SNHG3 group had the highest enrichment of miR-151a-3p (*P* < .001, Figure 3F). Furthermore, inhibition of SNHG3 significantly increased miR-151a-3p (*P* < .001, Figure 3G). Altogether, SNHG3 directly targeted miR-151a-3p and negatively regulated its expression in CCA.

### Inhibition of SNHG3 Suppressed the Aggressive Behavior of Cholangiocarcinoma Cells by Targeting miR-151a-3p

**Figure 4.**
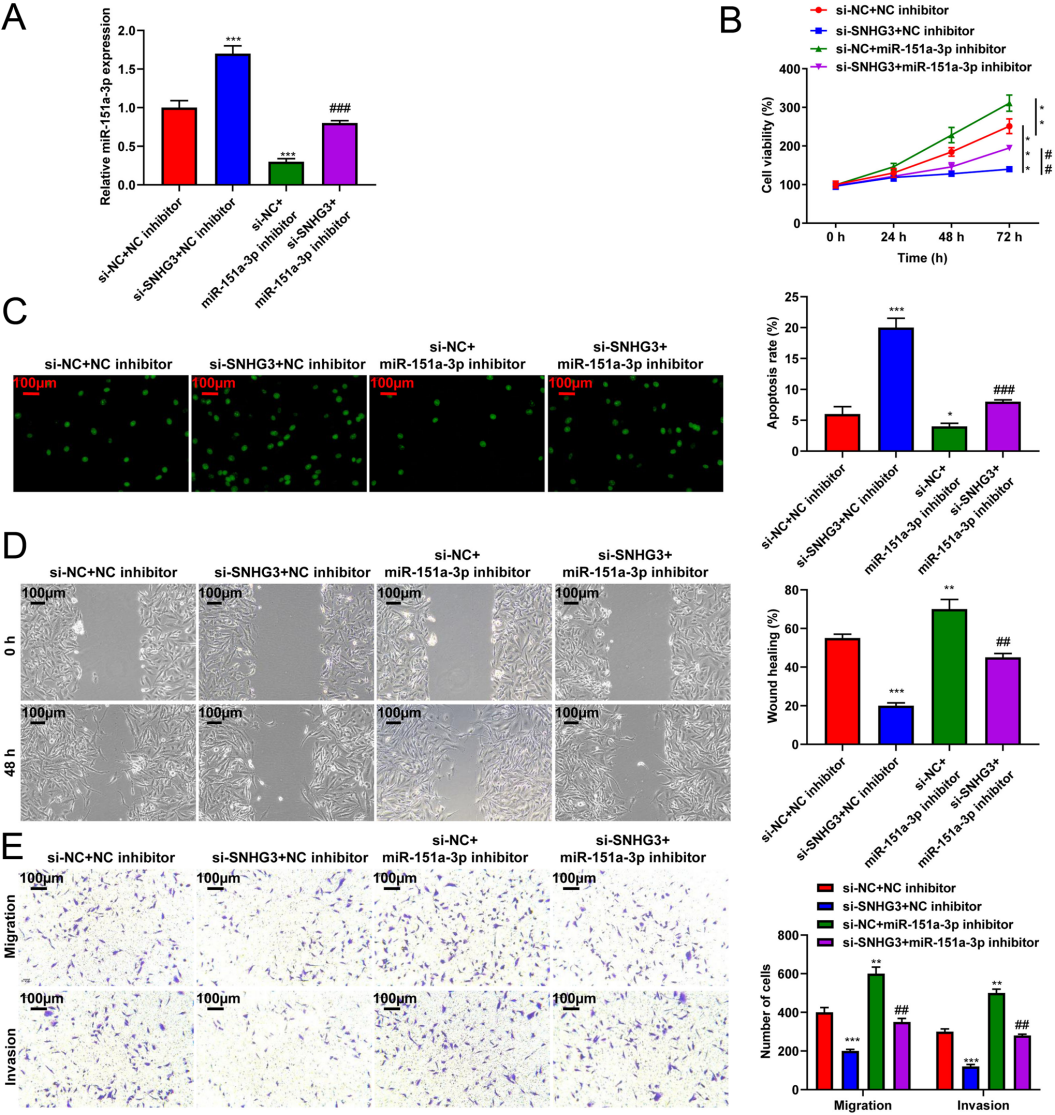
Inhibition of SNHG3 suppressed the aggressive behavior of CCA cells by targeting miR-151a-3p. HUCC-T1 cells were transfected with si-SNHG3 and miR-151a-3p inhibitor for 24 hours. (A) qRT-PCR assay was used to detect miR-151a-3p expression in HUCC-T1 cells. (B) CCK-8 assay was used to assess HUCC-T1 cells viability. (C) TUNEL was performed to detect HUCC-T1 cells apoptosis. (D) Wound healing assay was carried out to investigate HUCC-T1 cells migration (magnification: ×100). (E) Transwell assay was used to elucidate HUCC-T1 cells migration and invasion (magnification: ×100). ^*^P < .05, ^**^P < .01, ^***^P < .001 *vs*. si-NC+NC inhibitor; ^##^ *P* < .01, ^###^ *P* < .001 vs. si-SNHG3+NC inhibitor. CCA. cholangiocarcinoma; NC, negative control.

Based on the abovementioned results, the function of the SNHG3/miR-151a-3p axis in CCA development was investigated. Inhibition of SNHG3 increased miR-151a-3p, whereas treatment with a miR-151a-3p inhibitor downregulated miR-151a-3p in CCA cells (*P* < .001, Figure 4A). The miR-151a-3p inhibitor suppressed apoptosis (*P* < .001, Figure 4C) and increased the viability, migration, and invasive ability of HUCC-T1 cells transfected with si-SNHG3 (*P* < .01, *P* < .001, Figure 4B, D-E). The data collectively indicated that SNHG3 negatively regulated miR-151a-3p to promote the growth and metastasis of CCA.

### MiR-151a-3p Directly Targeted STAT5a in Cholangiocarcinoma

**Figure 5.**
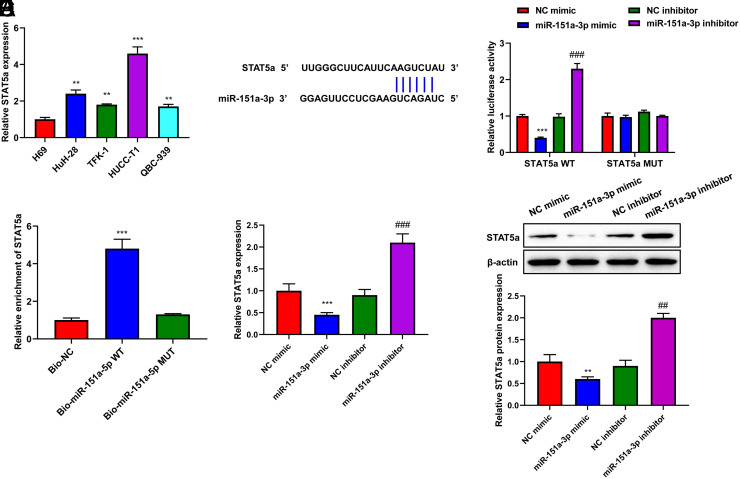
MiR-151a-3p directly targeted STAT5a in CCA. (A) The level of STAT5a in CCA cell lines (Huh-28, TFK-1, HUCC-T1, and QBC-939) and normal human cholangiocyte cell lines (H69) was measured by qRT-PCR assay. (B) The promising binding sites between STAT5a and miR-151a-3p were predicted by Starbase. (C) Dual-luciferase reporter assay was carried out to prove the relationship between STAT5a and miR-151a-3p. (D) RNA pull down assay was used to detect the binding situation between STAT5a and miR-151a-3p. (E) The mRNA level of STAT5a in HUCC-T1 cells with miR-151a-3p mimic miR-151a-3p inhibitor transfection was detected by qRT-PCR assay. (F) The protein level of STAT5a in HUCC-T1 cells with miR-151a-3p mimic or miR-151a-3p inhibitor transfection was detected by Western blot assay. ^**^P < .01, ^***^P < .001 *vs*. H69 or NC mimic; ^##^*P* < .01 vs. NC inhibitor. CCA, cholangiocarcinoma; NC, negative control.

STAT5a was highly expressed in CCA cell lines (*P* < .001, Figure 5A). The Starbase tool showed that the 3’-UTR of STAT5a could bind to miR-151a-3p (Figure 5B). The luciferase activity of STAT5a-WT was decreased by miR-151a-3p mimic, and elevated by miR-151a-3p inhibitor (*P* < .01, Figure 5C). RNA pull-down assay validated that biotinylated miR-151a-3p was bound to STAT5a (*P* < .01, Figure 5D). In addition, miR-151a-3p negatively modulated STAT5a expression (*P* < .001, Figures 5E-F). Altogether, miR-151a-3p directly targeted STAT5a to regulate its expression.

### Inhibition of SNHG3 Suppressed the Aggressive Behavior of Cholangiocarcinoma Cells Through the miR-151a-3p/STAT5a Axis

**Figure 6.**
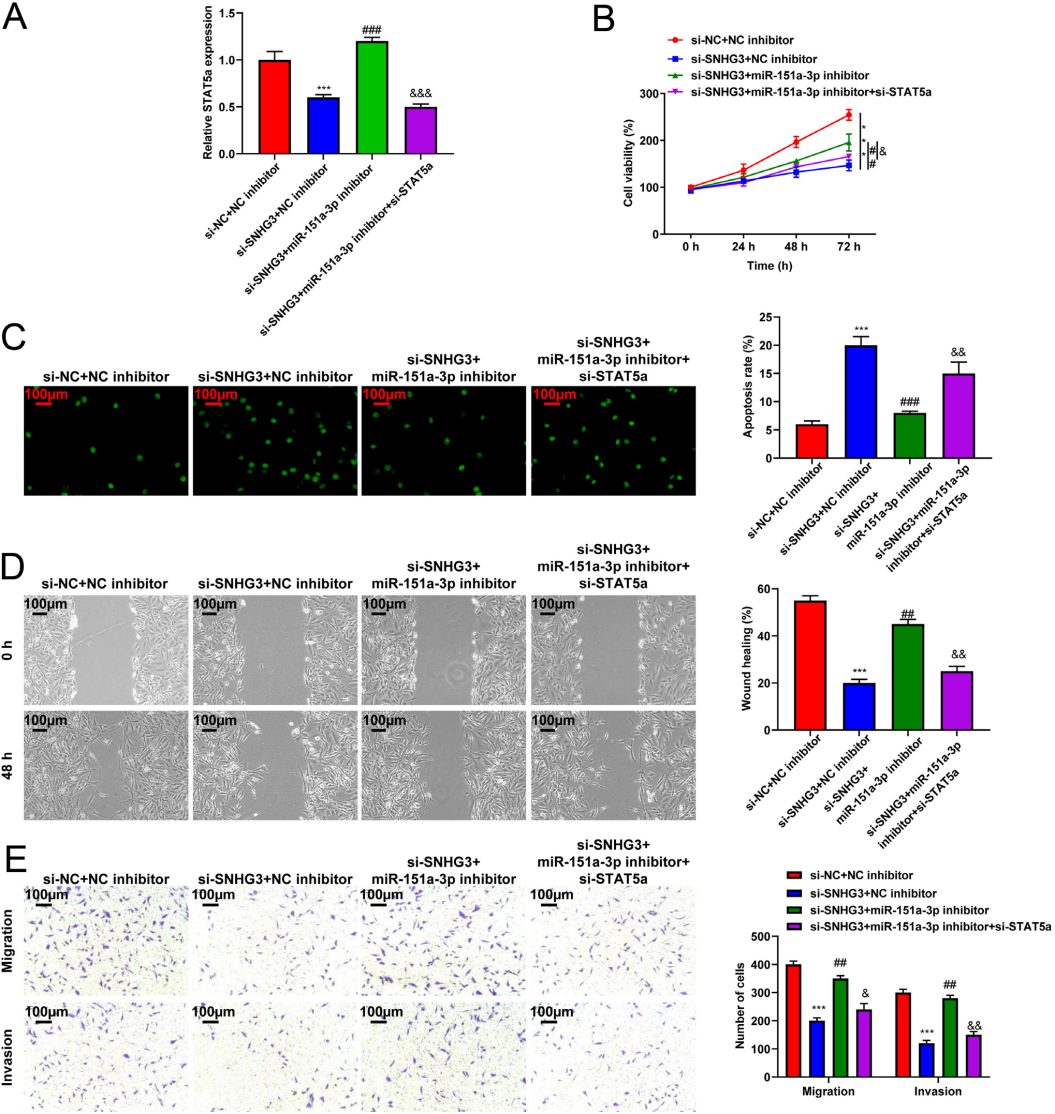
Inhibition of SNHG3 suppressed the aggressive behavior of CCA cells through the miR-151a-3p/STAT5a axis. HUCC-T1 cells were transfected with si-SNHG3, miR-151a-3p inhibitor and/or si-STAT5a for 24 hours. (A) qRT-PCR assay was used to detect STAT5a expression in HUCC-T1 cells. (B) CCK-8 assay was used to assess HUCC-T1 cells viability. (C) TUNEL was performed to detect HUCC-T1 cells apoptosis. (D) Wound healing assay was carried out to investigate HUCC-T1 cells migration (magnification: ×100). (E) Transwell assay was used to elucidate HUCC-T1 cells migration and invasion (magnification: × 100). ^***^P < .001vs. si-NC+NC inhibitor; ##*P* < .01, ###*P* < .001 *vs*. si-SNHG3+NC inhibitor; &*P* < .05, &&*P* < .01, &&&*P* < .001 vs. si-SNHG3+miR-151a-3p inhibitor. CCA, cholangiocarcinoma; NC, negative control.

The function of the SNHG3/miR-151a-3p/STAT5a axis in CCA progression was further investigated. qRT-PCR showed that STAT5a expression was lower in the si-SNHG3 + NC inhibitor group than in the si-NC + NC inhibitor group but higher in the si-SNHG3 + miR-151a-3p inhibitor group than in the si-SNHG3 + NC inhibitor group (*P* < .001, Figure 6A). The impact of SNHG3 and miR-151a-3p suppression on the viability, apoptosis, migration, and invasive ability of HUCC-T1 cells was counteracted by STAT5a knockdown (*P* < .001, Figures 6B-E). Altogether, SNHG3 promoted the growth and metastasis of CCA through the miR-151a-3p/STAT5a axis.

## Discussion

Surgical excision is the most effective treatment strategy for CCA. However, most CAA cases are diagnosed at an advanced stage, which makes radical surgery challenging. Therefore, identifying effective biomarkers is necessary to prevent the progression of CCA.

Long non-coding RNAs (LncRNAs) exhibit tissue- and disease-specific expression patterns.^14^ Studies have shown that lncRNAs hold promise in the diagnosis and prognosis of various cancers.^15,16^ For instance, the lncRNA H19 possesses excellent specificity and sensitivity as a diagnostic biomarker in gastric cancer.^15^ In CCA, abnormal expression of lncRNAs is closely associated with tumor size, differentiation, metastasis, and other clinicopathological parameters.^9,17^ Several lncRNAs, including CCAT, TUG1, CRNDE, and MNX1-AS1, have been identified as promising prognostic biomarkers for CCA.^18^ In addition, MIR4435-2HG and GAPLINC have been identified as hub lncRNAs that serve as promising biomarkers for CCA.^19^

LncRNAs are crucial in controlling the initiation and progression of various cancers, including tumor invasion and metastasis, and the acquisition of drug resistance.^20-23^ The lncRNA LYPLAL1-DT has been reported to attenuate cancer cell proliferation in breast cancer.^21^ Zhou et al. showed that the lncRNA MIR155HG was elevated in colorectal cancer and its increased level promoted M2 macrophage polarization and oxaliplatin resistance.^22^ In addition, the lncRNA FGD5-AS1 accelerated cancer cell metastasis.^23^ With regard to the effects of lncRNAs on CCA development,^24,25^ studies have shown that TTN-AS1 is upregulated and promotes tumorigenesis in CCA.^25^ The lncRNA ST8SIA6-AS1 targets miR-145-5p to increase the expression of MAL2, thereby promoting CCA development.^26^ In addition, HANR promotes the migration, proliferation, and stemness of CCA cells by binding to NICD and activating the Notch signaling cascade.^27^

Numerous malignancies have been linked to SNHG3 as an oncogene.^8,28,29^ For instance, Dai et al found that SNHG3 accelerated the development of bladder cancer,^28^ and Xie et al suggested that SNHG3 accelerated the metastasis of gastric cancer.^29^ In this study, CCA tissues and cells had elevated levels of SNHG3. Inhibition of SNHG3 suppressed the aggressive behavior of CCA cells, which is consistent with the findings reported by Sun et al.^30^

LncRNAs interact with miRNAs to regulate target genes.^26,31^ Their function as ceRNAs is one of the important mechanisms of post-transcriptional regulation of genes in the cytoplasm.^32^ miRNAs can modulate cancer cell function by directly targeting mRNAs and negatively regulating their expression.^33^ They are involved in various processes contributing to cancer development and progression.^34^ lncRNAs function as miRNA sponges to participate in tumor progression.^35^ The PRRT3-AS1/miR-507/HOXB5 pathway has been shown to enhance the oncogenic properties.^36^ The lncRNA MT1JP can alleviate the malignant behavior of CCA cells via the miR-18a-5p/FBP1 pathway.^31^ In addition, the lncRNA TTN-AS1 functions as a ceRNA of miR-320a, promoting cell cycle progression and angiogenesis in CCA.^25^ In osteosarcoma, SNHG3 binds to miR-151a-3p, increasing the malignant behavior of osteosarcoma cells.^8^ On the basis of these findings, we speculated that SNHG3 might mediate CCA progression by acting as a ceRNA. In this study, we initially found that SNHG3 was mostly distributed in the cytoplasm, which indicated its regulation at the post-transcriptional level. Subsequently, bioinformatic tools and mechanistic assays showed that SNHG3 directly targeted miR-151a-3p. Several types of malignancies show aberrant expression of miR-151a-3p, which is linked to tumorigenesis.^11,37^ For instance, miR-151a-3p suppresses prostate cancer cell growth.^11^ In addition, SBF2-AS1 sponges miR-151a-3p to induce chemotherapy resistance in tumor cells.^37^ It is unknown, therefore, how miR-151a-3p affects the development of CCA. miR-151a-3p was downregulated in CCA and its inhibition reversed the effects of SNHG3 inhibition on the aggressive behavior of CCA cells. To the best of our knowledge, this study is the first to demonstrate the role of miR-131a-3p in CCA progression.

In the study, miR-151a-3p and SNHG3 negatively and positively regulated the expression of STAT5a, respectively. In particular, miR-151a-3p negatively regulated STAT5a expression by directly binding to its 3’-UTR. Signal transduction and transcription activator (STAT) 5, a member of the STAT family, contains 2 isoforms, namely, STAT5s and STAT5b.^38^ Dysregulation of STAT5a has been shown to influence prognosis and the efficacy of radiotherapy and chemotherapy in various malignant tumors.^38-41^ Tan et al. discovered that STAT5A promoted glioma tumorigenesis by inducing LINC01198 to stabilize the RNA-binding protein DGCR8.^40^ Dong et al. found that STAT5a remodeled lipid metabolism in gastric cancer cells by regulating the FABP5 protein, consequently promoting gastric cancer tumorigenesis.^41^ Herein, STAT5a was found to be remarkably upregulated in CCA cells and tissues. Functional experiments validated that the effects of suppressing SNHG3 and miR-151a-3p on the aggressive behavior of CCA cells were counteracted by STAT5a knockdown.

These results suggest that SNHG3 facilitates the growth and metastasis of CCA through the miR-151a-3p/STAT5a axis. To the best of our knowledge, this study is the first to report the functional role of SNHG3 as a ceRNA of miR-151a-3p in CCA, its high expression in CCA tissues, and its significant association with clinicopathological parameters of CCA. The SNHG3/miR-151a-3p/STAT5a axis represents a promising therapeutic target for CCA.

Despite important findings, this study has some limitations that should be acknowledged. First, animal experiments are required to validate the oncogenic role of SNHG3 in CCA. Second, we selected miR-151a-3p as the target of SNHG3. However, other miRNAs may be involved in SNHG3-mediated development of CCA. To address these limitations in future studies, we will use animal models and molecular biology techniques to investigate the mechanism of SNHG3 in CCA development.

## Conclusion

In conclusion, this study reveals that SNHG3 is highly expressed in CCA tissues and promotes the aggressive behavior of CCA cells by targeting the miR-151a-3p/STAT5a axis. The novel axis comprising SNHG3, miR-151a-3p, and STAT5a is a promising therapeutic target for CCA.

## Availability of Data and Materials:

The data that support the findings of this study are available on request from the corresponding author.

## Supplementary Materials

Supplementary Material

## Figures and Tables

**Table 1.** Correlation Between SNHG3 Expression and Clinicopathological Characteristics

Variables	Total (30)	SNHG3 Low Expression (14)	SNHG3 High Expression (16)	*P*
Sex				.525
Male	17	8	9	
Female	13	6	7	
Age (years)				.437
≤60	12	5	7	
>60	18	9	9	
Tumor differentiation				.038
Well or moderate	10	7	3	
Poor	20	7	13	
TNM stage				.028
I/II	14	10	4	
III/IV	16	4	12	
Lymph node invasion				.032
Negative	15	8	7	
Positive	15	6	9	
Lymph node metastasis				.038
No	20	11	9	
Yes	10	3	7	

**Supplementary Table 1.** The Sequences for Cell Transfection

Gene	Sequences (5’-3’)
si-SNHG3	GGGAUCAUCUAGAAGGUAATT
si-STAT5a	TGATGGAGGTGTTGAAGAA
sh-NC	AAUUCUCCGAACGUGUCACGU
miR-151a-3p mimic	CUAGACUGAAGCUCCUUGAGG
miR-151a-3p inhibitor	CCGAAAGGAGAUUCAGUCUAG
NC mimic	UUUGUACUACACAAAAGUACUG
NC inhibitor	CAGUCCUUUUGUGUAGUACAA

**Supplementary Table 2.** Primer Sequences for qRT-PCR Assay

Gene	Forward Primer (5’-3’)	Reverse Primer (5’-3’)
SNHG3	GACTTCCGGGCACTTCGTAA	TGCTCCAAGTCTGCCAAAGA
STAT5a	GTGCCTGACAAAGTGCTGTG	GAGGTTCTCCTTGGTCAGGC
miR-196a	CCGACGTAGGTAGTTTCATGTT	GTGCAGGGTCCGAGGTATTC
miR-128	GGTCACAGTGAACCGGTC	GTGCAGGGTCC GAGGT
miR-1286	TGCAGGACCAAGATGAGCCCT	GCGAGCACAGAATTAATACGAC
miR-151a-3p	GGATGCTAGACTGAAGCTCCT	CAGTGCGTGTCGTGGAGT
GAPDH	GAGAAGGCTGGGGCTCATTT	AGTGATGGCATGGACTGTGG
U6	CTCGCTTCGGCAGCACA	AACGCTTCACGAATTTGCGT
