# Supplementary material for: Long Non-Coding RNA SNHG3 Promotes the Progression of Cholangiocarcinoma by Regulating the miR-151a-3p/STAT5a Axis
Source: Turk J Gastroenterol. 2024 Dec 1;35(12):933–44. doi: 10.5152/tjg.2024.24140 (PMC11639604; doi:10.5152/tjg.2024.24140)
Supplement: Supplementary Material [file supplementary_material.pdf]

Supplementary Table 1. The Sequences for Cell Transfection

Gene	Sequences (5′-3′)
si-SNHG3	GGGAUCAUCUAGAAGGUAATT
si-STAT5a	TGATGGAGGTGTTGAAGAA
sh-NC	AAUUCUCCGAACGUGUCACGU
miR-151a-3p mimic	CUAGACUGAAGCUCCUUGAGG
miR-151a-3p inhibitor	CCGAAAGGAGAUUCAGUCUAG
NC mimic	UUUGUACUACACAAAAGUACUG
NC inhibitor	CAGUCCUUUUGUGUAGUACAA

Supplementary Table 2. Primer Sequences for qRT-PCR Assay

Gene	Forward Primer (5′-3′)	Reverse Primer (5′-3′)
SNHG3	GACTTCCGGGCACTTCGTAA	TGCTCCAAGTCTGCCAAAGA
STAT5a	GTGCCTGACAAAGTGCTGTG	GAGGTTCTCCTTGGTCAGGC
miR-196a	CCGACGTAGGTAGTTTCATGTT	GTGCAGGGTCCGAGGTATTC
miR-128	GGTCACAGTGAACCGGTC	GTGCAGGGTCC GAGGT
miR-1286	TGCAGGACCAAGATGAGCCCT	GCGAGCACAGAATTAATACGAC
miR-151a-3p	GGATGCTAGACTGAAGCTCCT	CAGTGCGTGTCGTGGAGT
GAPDH	GAGAAGGCTGGGGCTCATTT	AGTGATGGCATGGACTGTGG
U6	CTCGCTTCGGCAGCACA	AACGCTTCACGAATTTGCGT
